# The Social Support Networks Scale (SSNS) for Family Caregivers of Children with Cancer: A Psychometric Evaluation

**DOI:** 10.3390/ijerph17217820

**Published:** 2020-10-26

**Authors:** Filiberto Toledano-Toledano, José Moral de la Rubia, René Reyes Frometa, Fabiola González Betanzos, Laura Villavicencio Guzmán, Marcela Salazar García

**Affiliations:** 1 Evidence-Based Medicine Research Unit, Hospital Infantil de México Federico Gómez National Institute of Health, Mexico City 06720, Mexico; 2 Facultad de Psicología, Subdirección de Posgrado, Universidad Autónoma de Nuevo León, Monterrey 64460, Mexico; jose_moral@hotmail.com; 3 Faculty of Psychology, Universidad Michoacana de San Nicolas de Hidalgo, Michoacán 58030, Mexico; renerf88@gmail.com (R.R.F.); fabiolagonzalezbetanzos@gmail.com (F.G.B.); 4 Developmental Biology Research Laboratory, Hospital Infantil de México Federico Gómez National Institute of Health, Mexico City 06720, Mexico; lauvillavi@gmail.com (L.V.G.); marcelasalazargarcia@hotmail.com (M.S.G.)

**Keywords:** social support, family caregivers, cancer, México, family functioning, quality of life, resilience

## Abstract

Currently, information about the psychometric properties of the Social Support Networks Scale (SSNS) for family caregivers of children with cancer is not yet available; therefore, there is no empirical evidence of its validity and reliability to support its use in this population. The aim of this study is to determine a factorial model of the SSNS, estimate its internal consistency reliability, describe its distribution, and check its concurrent validity. A convenience sample of 633 family caregivers of children with cancer hospitalized in a National Institute of Health in Mexico City was collected. The SSNS, a sociodemographic variables questionnaire, and three instruments that evaluated family functioning, quality of life, and resilience were applied. The five-factor model had a poor data fit and lacked discriminant validity. The sample was divided. In a subsample of 316 participants, exploratory factor analysis suggested a four-factor model. When testing the four-factor model through confirmatory factor analysis, religious support was independent of family support, friend support, and lack of support. In the other subsample of 317 participants, the one-factor model for religious support had a good fit, and the correlated three-factor model, with the remaining factors, showed an acceptable fit. Reliability ranged from acceptable (Guttman’s λ_2_ = 0.72) to good (λ_2_ = 0.88). Socio-family support and its three factors were correlated with family functioning, resilience, and quality of life. Religious support was correlated with four factors of resilience and quality of life. A scale of socio-family support with three factors and an independent scale for religious support are defined from the SSNS, and they showed internal consistency and construct validity.

## 1. Introduction

### 1.1. Pediatric Cancer as a Health Problem

Data from the World Health Organization (WHO) indicate that cancer is the second leading cause of death worldwide, where approximately one in six deaths is due to cancer in the total population, that is, including all ages [1]. On the other hand, in the population of children and adolescents, cancer is the main cause of death worldwide [1]. Nevertheless, in high-income countries, 80% of children diagnosed with cancer are cured and survive cancer [1]. The most common types of cancer are leukemia, brain cancer, lymphoma, and tumors such as neuroblastoma and Wilms’ tumor [1]. Mexico’s official figures indicate that the group from 0–4 years had the highest cancer incidence rate, adolescents between 15–19 years had the highest mortality rate, and the survival rate in children and adolescents was 57.5% [2].

In this context, the research literature has indicated that pediatric chronic diseases represent a life-changing event presenting an important challenge for families, their health, and coping [3]. These diseases have physical, psychological, socioeconomic, and behavioral effects that decrease quality of life and family functioning and increase the burden of the caregiver [4], all of which are aspects that define and structure the profile of adversity and vulnerability of families of children with chronic diseases [5].

### 1.2. The Family Caregiver of a Child with Cancer

In particular, the family caregiver of a child with cancer experiences various feelings and profound changes in the family’s daily life; children with cancer need to spend substantial time in hospitals, leading to the emotional, physical, mental, and socioeconomic exhaustion of the caregiver [6]. In addition, parents experience life stress associated with constant hospital admissions and patient care [7].

In the research literature, a family caregiver is defined as a person who has a significant emotional bond with the patient. This caregiver is a family member who is part of the patient’s family life cycle, offers emotional-expressive and instrumental support, and provides comprehensive care and assistance during the chronic illness, acute illness, or disability of a child, adult, or elderly person [8].

Family caregivers can be affected physiologically and psychosocially by experiencing depression [8], anxiety [9], and posttraumatic stress [10]. All of these psychosocial variables have consequences that involve risk factors for developing psychopathology and caregiver burden [4].

However, despite the adversity, risk, and vulnerability experienced by families of children with cancer, there are protective factors that can reduce the negative impact of care. These factors can generate adaptive responses in families and in their psychosocial functioning to overcome the psychosocial consequences of caring for children with cancer. Among the protective factors are coping styles [3], resilience [11,12], and, most critically, perceived social support, that is, social support networks through family, friends, neighbors, religion, and governmental and nongovernmental institutions [13].

### 1.3. Effect of Social Support on Family Caregivers of Children with Cancer

Social support refers to a social network’s provision of psychological and material resources intended to benefit an individual’s ability to cope with stress. It is often differentiated in terms of three types of resources: instrumental, informational, and emotional [14]. The type of information given leads persons to think that they are esteemed, loved, and cared. This support is usually provided in a situation of illness or during a stressful event and protects people in crisis from a variety of psychopathological states [15]. It is usually defined as the existence or availability of people on whom we can rely, and who let us know that they care about, value, and love us [16].

In this regard, family, friends, and neighbors may bolster the social support perceived by family caregivers, influencing their daily life through cohesion, open communication, and creating a positive environment to care for the child with cancer [17].

With respect to the theoretical links between social support, health, and well-being, empirical evidence shows that being able to rely on a social support network in times of adversity contributes to overall health; on the other hand, when people perceive a lack of support or a diminishing social support network, they can become emotionally unstable and pessimistic [18]. In particular, the lack of social support in contexts of illness can affect the physical and mental health of family caregivers and have emotional and behavioral consequences [19,20,21,22,23,24].

### 1.4. Instruments for Measuring Social Support

The international literature has reported a set of measurement instruments that evaluate social support in various contexts, populations, and cultures. Most of them have been subjected to validation processes and cross-cultural adaptation. To the best of our knowledge based on bibliographic reviews, none have been validated and adapted in the context of Mexican family caregivers of children with cancer; therefore, it is not possible to adequately extrapolate these measurement instruments to this population because there is no empirical evidence of its validity and reliability to support its use in this population.

Several studies have confirmed that the psychometric properties of instruments measuring social support are consistent over time. One study in Spain evaluated the validity and reliability of the Duke-UNC-11 instrument, which measures functional social support [25]. In this study, 656 people were interviewed in a health center. The intraclass correlation coefficient of the 11 items was greater than 0.50 between the two formats of administering the questionnaire. Its internal consistency was excellent (α = 0.92) for the self-report format and good (α = 0.80) when administered by an interviewer; it had a two-factor structure, and the multiple linear regression explained 30% of the variance in social support through family functioning, education level, perceived internal control of health, mental health, and perception of disease severity. Therefore, it showed reliability and construct validity in that population.

In another study developed to validate the Medical Outcomes Study-Social Support Survey (MOS-SSS) questionnaire at a Spanish cancer institute, the instrument was applied to a population of 400 outpatients. Exploratory factor analysis (EFA) extracted three factors: emotional-informational support, affective support, and instrumental support. The overall internal consistency was excellent (α = 0.94). The authors concluded that the MOS-SSS is a valid instrument to measure perceived social support [26].

Other researchers, Dambi et al. [27], conducted a psychometric evaluation of the Multidimensional Scale of Perceived Social Support Scale (MSPSS) in family caregivers of cancer patients in Zimbabwe. In this study, 126 families were interviewed. The results of the EFA and the confirmatory factor analysis (CFA) showed evidence of structural validity for the two-factor model. In addition, the overall internal consistency was excellent (α = 0.90).

In addition, a study was conducted in the United States with the objective of psychometrically evaluating the Family Support Scale (FSS) developed by Littlewood et al. [23]. The FSS was applied to 255 family caregivers. The results of the EFA, using principal components and varimax rotation, showed a four-factor model: partner or spouse support, formal professional support, informal community support, and family support. The explained variance was 50%, and the overall internal consistency was acceptable (α = 0.79). The authors confirm that the FSS is a valid and reliable instrument for measuring social support in family caregivers [23].

### 1.5. Instruments for Measuring Social Support in Mexico

In Mexico, research regarding social support and the evaluation thereof have been developed through the design and development of valid, reliable, and culturally relevant measurement instruments. Among these instruments is the Scale of Social Support in Mexican Adults, which was designed using a natural semantic networks technique in its qualitative phase. In the quantitative phase, EFA obtained a scale of 26 items distributed in four factors: emotional, companionship, validation, and practical-instrumental. These factors explained 56% of the variance, and its overall reliability coefficient was excellent (α = 0.93). Therefore, it was determined to be a valid, reliable, and culturally relevant scale to evaluate social support in the general Mexican population [18].

Martínez et al. [28] conducted a study in Mexican patients with HIV with the aim of validating the Medical Outcomes Study (MOS) of Sherbourne and Stewart [29]. The Spanish translation by De la Revilla et al. [30] was used. A total of 313 adult patients from a National Institute of Health of Mexico City participated. Through the principal component method and varimax rotation, EFA revealed two factors: emotional and informational support (α = 0.97) and tangible support (α = 0.89). Both factors explained 72% of the variance. In addition, the two-factor structure presented a good data fit according to the CFA [28].

Another instrument is the Quality of Networks Scale (QNS) by Nava-Quiroz [31]. The QNS emerged as a result of a literature review on social support. The main findings of the review indicated a set of theoretical and methodological limitations in the published studies. One limitation was that most instruments had been developed with the objective of assessing the contribution of the social environment to the field of health and had not considered measuring or evaluating social support networks. In addition, most of these instruments had been designed based on the author’s subjective view about the construct but lacked content and social support construct validation [31].

Based on these gaps in the research literature, Nava-Quiroz [31] designed, constructed, and validated the QNS, which evaluates perceived network quality focusing on cohesion, communication, trust, help, and mutual respect. After developing the items from a panel of experts, the scale was applied to 215 psychology students. The EFA, through principal component analysis and varimax rotation, revealed three conceptually clear factors: family bond with 15 items (α = 0.94), friendship and trust with 14 items (α = 0.91), and neighbor-peers with 6 items (α = 0.69). The scale, reduced to 35 items, had excellent overall reliability (α = 0.92), and these three factors explained 48.1% of the variance [31].

In Mexico, studies have been conducted to test the psychometric properties of the QNS in the general population [32]. In this study, 200 inhabitants of the city of Morelia with an age range of 21 to 70 years participated. After a theoretical and empirical study, López-Peñaloza [32] added 10 items to the QNS that aim to measure more specific aspects of Mexican culture. With the 45 items, she obtained five factors: support from friends (α = 0.91), family support (α = 0.90), lack of support (α = 0.77), religious support (α = 0.86), and support from neighbors (α = 0.78), which explained 52.03% of the variance. The overall internal consistency was excellent (α ± 0.92). The resulting new scale was called the Social Support Network Scale (SSNS). These results of the psychometric properties of the SSNS have been useful in subsequent studies on family functioning and its relationship with social support networks [33].

Although the QNS and SSNS have been applied to university students, the general population and parents, to date, there is no evidence that they have been used and validated in populations of family caregivers of children with cancer. It should be noted that the SSNS has broader cultural content for the Mexican context and better metric properties than the QNS; therefore, the SSNS was chosen in the present study.

### 1.6. Problem Statement

Research regarding the impact of social support on family caregivers is promising, but one of its limitations is having reliable measurement instruments that have been validated in this specific population. The SSNS scale can be useful for this purpose and has the advantage of having been developed in the Mexican cultural context. However, it should be considered that this test was originally validated for use in the general population; therefore, its use in other populations, such as family caregivers of children with cancer, would compromise the validity and reliability of its results, due to the lack of psychometric data.

In response to this need, the aim of the present study was to analyze psychometric properties of the SSNS. To this end, we stated four objectives: (1) to test the five-factor model proposed by López-Peñaloza [32]; (2) to estimate the internal consistency reliability of the scale and its factors; (3) to describe their distributions; and (4) to prove the concurrent validity of the SSNS in relation to family functioning, quality of life, and resilience among family caregivers of children hospitalized with cancer in a National Institute of Health in Mexico City.

The expectations were as follows: the five-factor model would have a good or acceptable fit to the data, as evidence of structural construct validity; the scale would show excellent internal consistency reliability, with the five factors ranging from excellent to acceptable [32,33]; the distributions of the scale and its factors would follow a nonnormal distribution with negative skewness because most of the participants usually report that they receive very much support and a minority of people usually report that they receive very little support [32,34]; and social support would be positively correlated with family functioning [17,33], resilience [35], and quality of life [36], as evidence of concurrent construct validity.

## 2. Materials and Methods

### 2.1. Participants

Nonprobabilistic sampling was performed. The convenience sample consisted of 633 family caregivers of children with cancer hospitalized at a National Institute of Health in Mexico City. The inclusion criteria for this study were as follows: being a family caregiver of a child with chronic illness hospitalized in the institution, being older than 18 years, and signing an informed consent form. The sample met the minimum criteria of participants (*n* > 200) to perform EFA [37] and CFA [38]. An initial CFA was performed with the total sample of 633 caregivers. The sample was then subdivided into 2 groups. In sample A, with 316 caregivers, the factorial structure was explored, and in sample B, with 317 caregivers, the new factorial model was tested. The analyses for reliability, description of the sample, and convergent validity were performed for the total sample of 633 participants. The Results section describes the total sample.

### 2.2. Measuring Instruments

A sociodemographic variables questionnaire for research on family caregivers of children with chronic diseases (Q-SV) by Toledano-Toledano et al. [39]: this questionnaire contains 20 questions that evaluate social, family, and clinical variables of the patient, for example, age and sex of the patient and caregiver, diagnosis, amount of time hospitalized and time since diagnosis, family role of the caregiver (mother, father, or other family member), education, occupation, marital status, years married, number of children, family type, family life cycle, support networks, religion, and monthly household income.

Social Support Network Scale (SSNS) by López-Peñaloza [32]: this is a self-report instrument in which each item is scored on a 5-point Likert scale, ranging from 1 (strongly disagree) to 5 (strongly agree). The items of the original scale are distributed in 5 factors: support from friends (15 items: 2, 5, 6, 10, 13, 17, 18, 27, 30, 31, 34, 38, 39, 42, and 45), family support (15 items: 1, 3, 7, 8, 9, 12, 14, 20, 21, 33, 37, 40, 41, 43, and 44), lack of support (7 items: 15, 22, 23, 25, 26, 29, and 35), religious support (4 items: 4, 16, 28, and 36), and support from neighbors (4 items: 11, 19, 24, and 32). Its overall internal consistency was excellent, α = 0.92 [32].

Measurement scale of Resilience in Mexicans (RESI-M) by Palomar and Gómez [40]: this instrument has been validated in family caregivers of children with cancer, by Toledano-Toledano et al. [11]. It is scored on a 4-point Likert scale, ranging from 1 (strongly disagree) to 4 (completely agree). The 43 items are distributed across 5 factors: strength and self-confidence (19 items: 1–19), social competence (8 items: 20–27), family support (6 items: 28–33), social support (5 items: 34–38), and structure (5 items: 39–43); overall internal consistency was excellent, α = 0.93 [11,12].

Family Functioning Scale (FFS) by García-Méndez et al. [41]: this instrument scores items on a 5-point Likert scale, ranging from 1 (never) to 5 (always). The 45 items are grouped into 4 factors: positive family environment (7 items: 12, 14, 15, 17, 18, 21, and 22), cohesion/rules (5 items: 1, 6, 9, 10, and 19), hostility/avoidance of conflict (5 items: 2, 3, 5, 16, and 20), and command/problems in expression of feelings (5 items: 4, 7, 8, 11, and 13). Good overall internal consistency was found, α = 0.89 [41].

World Health Organization Quality of Life Assessment (WHOQOL-BREF): this instrument was developed by the WHOQOL Group [42], belonging to the WHO program on mental health, and consists of 26 items that are scored using a 5-point Likert scale, ranging from 1 (very dissatisfied) to 5 (very satisfied). It was adapted for Mexico by González-Celis and Sánchez-Sosa [43]. The items are distributed across 4 factors: physical health (7 items: 3, 4, 10, 15, 16, 17, and 18), psychological (6 items: 5, 6, 7, 11, 19, and 26), social relationships (3 items: 20, 21, and 22), and environment (8 items: 8, 9, 12, 13, 14, 23, 24, and 25). In addition, item 1 evaluates overall perceived quality of life, and item 2 evaluates overall perceived health status; good overall internal consistency was found, α = 0.89 [43].

### 2.3. Procedure

Family caregivers were interviewed by the first author of this work, and the interviews were conducted from September 2019 to February 2020 in the children’s rooms in the oncology ward of Federico Gómez Children’s Hospital of Mexico at the National Institute of Health. All the family caregivers interviewed were invited to participate voluntarily, the objectives of the research were explained, and all their questions related to the study were answered. Those family caregivers who decided to participate signed an informed consent form and answered the questionnaires themselves in a single session.

### 2.4. Ethical Considerations

This study is a part of the Research Project HIM/2015/017/SSA.1207 “Effects of mindfulness training on psychological distress and quality of life of the family caregiver”, which was approved on December 16, 2014, by the Research, Ethics, and Biosafety Commissions of the Hospital Infantil de México Federico Gómez National Institute of Health, in Mexico City. While conducting this study, the ethical rules and considerations for research with humans currently enforced in Mexico [44] and those outlined by the American Psychological Association [45] were followed. All family caregivers were informed of the objectives and scope of the research and their rights according to the Helsinki Declaration [46]. The caregivers who agreed to participate in the study signed an informed consent letter. Participation in this study was voluntary and did not involve payment.

### 2.5. Statistical Analysis

Statistical calculations were performed with SPSS version 24 (IBM; Armonk, NY, USA), AMOS version 16 (IBM; Armonk, NY, USA), and Excel 2007 (Microsoft; Redmond, WA, USA). For the first objective of determining the factorial structure, the five-factor model of López-Peñaloza [32] was contrasted with the total sample of 633 participants. Since the population was different from the population in which the SSNS was developed and the model was poorly fit to the data and presented problems of discriminant validity between 2 factors, it was decided to explore the factorial structure and contrast the resulting model.

For these analyses, the recommendations that suggest randomly dividing the sample into 2 groups were followed [47]. In a subsample of 316 participants, the exploratory part of the analysis was performed, and in the other subsample of 317 participants, the confirmatory part of the analysis was performed.

The number of factors for the EFA was determined using the new empirical Kaiser criterion [48] and Horn’s [49] parallel analysis. The factors were extracted using the maximum likelihood (ML) estimation method in accordance with the method used for the CFA. Oblique rotation was applied to interpret the factors by the Promax method. All items with loads <0.40 in the structural matrix were eliminated. In addition, the one-dimensionality of each factor was analyzed using the same procedure; in cases where the items revealed a second factor, they were eliminated one by one, starting with the item with the greatest weight in the second factor.

In the CFA, the parameters were estimated using the ML method. Since multivariate normality was not strictly fulfilled, the bias-corrected percentile (BCP) method was used to estimate 95% confidence intervals and the significance of parameters. The likelihood-ratio chi-square test (χ^2^) was used to evaluate the fit of each model to the data. However, this index is sensitive to sample size. Consequently, other fit indices were taken into account, such as relative chi-square (χ^2^/df), standardized root mean square residual (SRMR), goodness of fit index (GFI), comparative fit index (AGFI), and Tucker–Lewis index (TLI), as well as the root mean square error of approximation (RMSEA) and its 90% confidence interval (90% CI). Acceptable data fit was defined according to the following criteria: χ^2^/df < 3, RMSEA < 0.08, SRMR < 0.10, GFI > 0.90, AGFI > 0.85, TLI > 0.90, and CFI > 0.90. Close data fit was defined according to the following criteria: χ^2^/df < 2, RMSEA < 0.05, SRMR < 0.05, GFI > 0.95, AGFI > 0.90, TLI > 0.95, and CFI > 0.95 [38,50].

To improve the model fit, a data-driven strategy was employed [51], and the model was redefined based on the modification indices that indicated a significant reduction in the chi-square value. It was verified that the modifications were justified; for example, in the cases in which covariance existed between the errors, redundancy in the content was analyzed, and the item with the smallest factorial weight was eliminated. This first analysis, still exploratory in nature, was performed in the same subsample A, with 316 participants, in which the EFA was performed. In addition, in this sample, 3 models were compared: the original five-factor model, the model derived from the four-factor EFA, and the model modified with the CFA data.

This last model was cross-validated by performing CFA for the 317 participants in the second sample. In addition, the convergent validity of each measurement model (degree of certainty that the items are measuring the same construct) was estimated through 3 criteria: measurement weights (*l*), composite reliability (McDonald ω), and average variance extracted (AVE). An acceptable level of convergent validity was attained when the average of *l* ≥ 0.50; ω ≥ 0.70; and AVE ≥ 0.37 for 4 items, ≥0.28 for 6 items, and ≥0.25 for 7 or more items. Good convergent validity implied *l* ≥ 0.50; ω ≥ 0.80; and AVE ≥ 0.50 for 4 items, ≥0.40 for 6 items, and ≥0.25 for 12 or more items [52]. The discriminant validity between factors (degree of certainty in 2 factors not sharing the same indicators) was established by a shared variance (r ^2^) < 0.81 and < AVE of each factor [53].

For the second objective, estimating reliability, the Guttman’s lambda-2 coefficient (λ_2_) was used because the assumption of tau-equivalence was not met, as revealed by unequal measurement weights within each factor. Values of λ_2_ < 0.50 were considered unacceptable reliability, between 0.50–0.59 poor, between 0.60–0.69 questionable, between 0.70–0.79 acceptable, between 0.80–0.89 good, and ≥0.90 excellent [54].

In relation to the third objective, describing the distributions, measures of central tendency (mean and median), variation (standard deviation and semi-interquartile range), shape of the distribution (coefficients of asymmetry and kurtosis based on central moments), and noncentral position (quartiles and deciles) were calculated. Normality was verified by the D’Agostino–Pearson and Kolmogorov–Smirnov tests with Lilliefors correction for calculating probability. Friedman’s test was used to compare averages of the factors. The effect size was estimated by Kendall’s W coefficient, and pairwise post hoc comparisons were performed via the Dunn–Bonferroni test.

For the fourth objective, concurrent validity, the correlations were calculated via the Spearman’s correlation coefficient. As in the previous objective, nonparametric tests were used because the distributions of the scores in the 2 scales (socio-family support and religious support) and their factors did not follow a normal probability distribution [55].

## 3. Results

### 3.1. Sociodemographic Characteristics of Family Caregivers of Children with Cancer

Table 1 shows the frequencies and percentages of the sociodemographic variables belonging to the 633 caregivers who participated in the study. Additionally, Table 1 provides information on sociodemographic and clinical variables of cancer patients.

### 3.2. Determining the Factorial Structure

#### Testing the Hypothetical Five-Factor Model

First, the hypothetical five-factor model, developed by López-Peñaloza [32], was tested. When estimating the parameters by ML and their significance by BCP in the total sample of 633 participants, the fit to the data was poor for most of the indices (Table 2). All parameters were significant, except for the correlation between lack of support (F3) and religious support (F4): r_F3,F4_ = 0.016, BCP CI 95% (−0.097, 0.132), *p* = 0.777. Neighbor support had questionable reliability (λ_2_ = 0.606) and lacked convergent validity (AVE = 0.269 and ω = 0.576). Another additional problem with this model was the single correlation between friend support (F1) and religious support (F4), r_F1,F2_ = 0.953, BCP 95% CI (0.901, 1.005), *p* < 0.001. Consequently, the shared variance between these two factors was greater than the variance extracted from each factor (r_F1,F2_^2^ = 0.908 > AVE_F1_ = 0.414 and AVE_F4_ = 0.474); therefore, there was no discriminant validity between the two factors (Table 3).

### 3.3. Exploration of the Factorial Structure

In subsample A, with 316 participants, there were four referenced eigenvalues greater than 1 and less than the empirical eigenvalues. Table 4 shows the arrangement obtained by extracting four factors and eliminating the items with factorial weights below 0.40 or defining a second factor when factorized individually. Horn’s parallel analysis indicated six factors, but two factors had only two items with loads greater than 0.40 in the matrix. With the 33 items retained, both criteria to determine the number of factors converged into four (Table 4).

**Figure 1:**
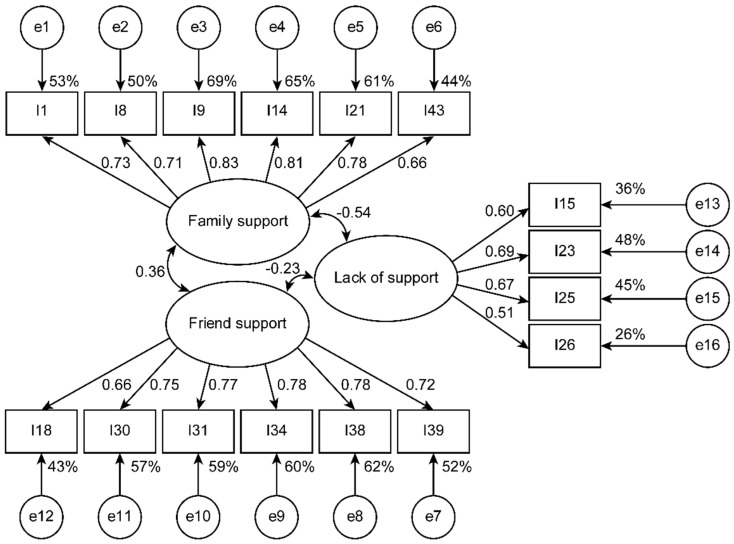
Adjusted 3-factor model estimated using subsample B (*n* = 317).

The fit of the four-factor model specifying 33 items, derived from the EFA, was improved with respect to the original model (Δχ^2^ [446] = 1096.632 *p* < 0.001; ΔTLI = 0.064, and ΔCFI = 0.064) but did not reach an acceptable fit (Table 2). This model revealed that religious support is independent of the other three factors (family support r = 0.034, BCP 95% CI (−0.109, 0.212), *p* = 0.606; support from neighbors r = −0.002, BCP 95% CI (−0.146, 0.175), *p* = 0.972; and lack of support r = 0.090, BCP 95% CI (−0.085, 0.248), *p* = 0.304). Items with high correlated residuals and lower weights were eliminated to improve the fit. Following this procedure, family support was reduced to six indicators (items 1, 8, 9, 14, 21, and 43), friend support was reduced to six indicators (items 18, 30, 31, 34, 38, and 39), lack of support was reduced to four indicators (items 15, 23, 25, and 26), and religious support was reduced to four indicators (items 4, 16, 28, and 36) (Figure 1). In this way, all indices achieved an acceptable fit (Table 2). When the religious support factor remained independent (i.e., from family support r = 0.015, BCP 95% CI (−0.121, 0.164), *p* = 0.769; support from neighbors r = −0.013, BCP 95% CI (−0.174, 0.154), *p* = 0.787; and lack of support r = 0.098, BCP 95% CI (−0.046, 0.307), *p* = 0.210), we decided to define a three-factor model for socio-family support or trusted relationships with 16 items and a single-factor model with four indicators for religious support. Consequently, the SSNS was divided into a 16-item socio-family support scale (SFS-16) or support from trusted relationships and a 4-item religious support scale (RSS-4).

**Figure 2:**
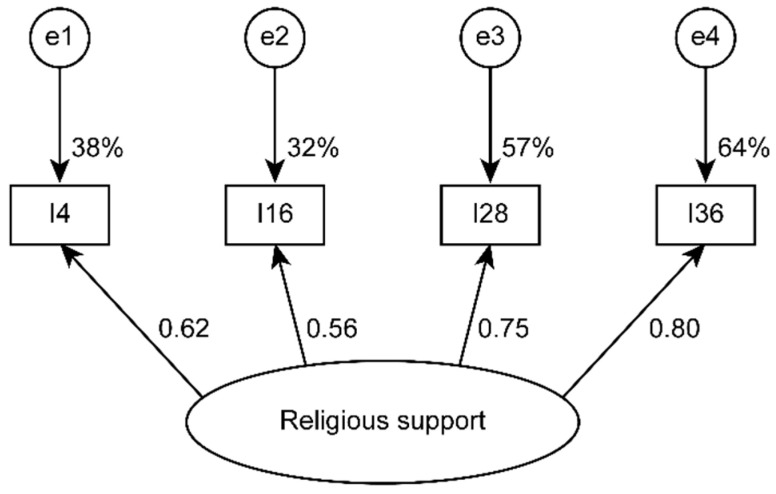
Single-factor model for religious support estimated using subsample B (*n* = 317).

In validation subsample B, with 317 participants, the three-factor socio-family support model with 16 items (SFS-16) had an acceptable fit (Table 2 and Figure 1), and the single-factor model for religious support with four items (RSS-4) showed a good fit (Table 2 and Figure 2). The three factors of the first model had convergent validity (good for FAS and FRS and acceptable for LS) and discriminant validity (Table 3). The religious support scale (RSS-4) presented acceptable convergent validity (Table 3).

**Figure 3:**
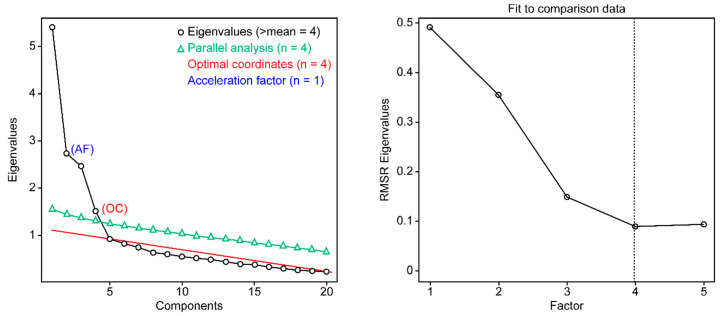
Determining the number of factors to retain through 5 empirical methods for the validation of the 2 new models: 16-item socio-family support scale (SFS-16) and 4-item religious support scale (RSS-4) (subsample B, *n* = 317).

Notably, if the number of factors in the validation subsample with 317 participants is determined, the number of factors converges to four, not only by the new empirical Kaiser criterion and Horn’s parallel analysis but also by other criteria such as optimal coordinates, minimum average of the squared partial correlations, and Ruscio and Roche’s comparison data approach (Figure 3).

### 3.4. Reliability of the SSRS in Its Final Version (SFS-16 and RSS-4)

Table 3 shows the internal consistency reliability analyses for the total items in each model and the factors in the original model and the two final models (SFS-16 and RSS-4). Calculations were made for the total sample of 633 participants. The reliability for the 16 items of the socio-family support scale (SFS-16) and the 4 items of the religious support scale (RSS-4) were acceptable. The reliability of the family support factors (FAS-6) and friends support factors (FRS-6) were good, and that for lack of support was acceptable (LS-4).

### 3.5. Description of the Distributions

Table 4 shows the descriptive statistics of the 16-item socio-family support scale (SFS-16) and its three factors (family support with six items (FAS-6), friend support with six items (FRS-6), and lack of support with four items (LS-4)), as well as the religious support scale with four items (RSS-4). The scores were obtained as the ratio between the sum of the items and the number of items added (average score); therefore, the potential range varies along a continuum from 1–5. The distributions of the scores in the two three-factor scales showed asymmetry and leptokurtosis for the SFS-16 and FAS-6. None followed a normal distribution. By dividing this range into five equal intervals corresponding to the five ordered response categories, it can be interpreted that scores between 1–1.79 indicate (average) strong disagreement with receiving support, that scores between 1.8–2.59 indicate disagreement, that scores between 2.6–3.39 neither agree nor disagree, that scores between 3.4–4.19 agree, and that scores between 4.2–5 strongly agree.

The medians for the SFS-16 and the religious support scale reflect support (agree). The median of the family support factor reflected strong support (strongly agree), that of friend support was ambiguous (neither agree nor disagree), and congruently, the median rating for lack of support was disagreement (Table 5).

When comparing the averages between the three factors of the SFS-16 (FAS-6, FRS-6, and LS-4) and the religious support scale (RSS-4) by Friedman’s test, there was a significant difference, χ^2^ (3, *n* = 633) = 1065, *p* < 0.001, with a very large effect size (Kendall’s W = 0.837). Dunn–Bonferroni post hoc pairwise comparison tests revealed that the averages could be ordered in the following sequence of significant differences: LS-4 < FRS-6 < RSS-4 < FAS-6 (Table 5).

### 3.6. Concurrent Validity

Table 6 shows the correlations of the three factors for the socio-family support scale (FAS-6, FRS-6, and LS-4) and the religious support scale (RSS-4) with the family functioning, quality of life, and resilience scales. These correlations correspond to the concurrent validity analysis.

The SFS-16 scale and its three factors had significant correlations with the convergent validity criteria, except for LS-4 with the structure factor of the resilience scale. The family support factor (FAS-6) had the highest correlations, and the friend support factor (FRS-6) had the lowest. Consistent with expectations, the correlations for the total score (SFS-16) and the family support factor (FAS-6) and friend support factor (FRS-6) had signs opposite to that of the lack of support factor (LS-4).

The religious support scale (RSS-4) showed a different correlation pattern. Almost all were not significant, except for positive correlations with social support and social competence factors in the resilience scale and negative correlations with environment and physical health factors in the quality of life scale. The first three correlations had a small strength of association, and the last had a negligible strength of association.

Table 6 shows the internal consistency reliability values of these scales and their factors in the total sample of 633 participants (SSNS and for RESI-M, FFS, and WHOQOL-BREF).

## 4. Discussion

The first objective of this research was to determine the factorial structure of the 45-item SSNS. The expectation was five correlated factors [32]. The model of five correlated factors was not validated. The neighbor support factor had reliability problems and was not distinguishable from the religious support factor. When exploring the factorial structure, the neighbor support factor was not defined. Therefore, neighbor support does not seem to be a relevant factor for family caregivers of children with cancer. In urban settings, especially in large cities, such as Mexico City, disconnection between neighbors is frequent [56], hence this factor is probably problematic in other populations residing in large cities. Nevertheless, the fact that neighbor support cannot be distinguished from religious support, when the latter is independent of family and friend support in the final version of the scale and presents a different correlation pattern, may indicate that the support of neighbors is not supportive in the instrumental and emotional sense but, rather, the ideological, spiritual, or imaginary sense. The family caregiver could receive intangible support by talking with their neighbors (remembering the strength they gain from their faith in God, “the tests the Lord puts in our path”, “his/her mysteries”, the value of the sacrifice) and participating with them in prayer activities in the church, Las Posadas over Christmas, preparations for the Guadalupe procession, or setting up altars for the dead.

The exploration of the factorial structure showed the expected factors: family support, friend support, lack of support, and religious support. The CFA showed that religious support is independent of the other three factors. Consequently, the SSNS was separated into two independent scales. The first scale evaluates support from family and friends (from work and outside of work), that is, support from bonding relationships that probably provide instrumental and emotional resources to cope with the child’s chronic illness. This scale was called the socio-family support scale or trusted relationships scale. One of its three factors is composed of direct family support items, another of direct friend support items, and a third of inverse items pertaining to a lack of support from family and friends. The second scale evaluates religious support. Its content includes support in faith in God, in religious beliefs, in attending church, and in prayer groups. There is no specific reference to neighbors, but they must be involved in prayer groups; therefore, it is no surprise that one of the items of the neighbor support factor refers to a religious holiday in December (Las Posadas).

One might wonder if the same results from independence of religious support would be expected in areas where one religion is not dominant and even religion does not represent significant support for many people. In the study by Medellín-Fonte et al. [33] on family functioning and its relationship with social support networks, carried out in Morelia, Mexico, religious support was associated with the support from friends, but not with family support, which is consistent with the present results. Religious people tend to seek friends with their beliefs and values [57,58], hence it is likely that this result is also seen in cities with a lot of religious diversity and a high percentage of atheists or people without religion. Given that this is not the case of Mexican cities [59], this hypothesis could be tested in cities, such as London, Potsdam, or Turin, where this diversity occurs [60,61].

The SSNS was reduced from five to four factors, three intercorrelated and one independent, that is, two scales. In addition, it was reduced from 45–20 items: 16 items for the socio-family support or trusted relationships scale and 4 items for the religious support scale. This reduction in items was done essentially to achieve a good fit, improve the convergent validity of the indicators, and preserve good or acceptable reliability. However, a concern that may arise as a result of this reduction in items is content validity.

The analysis strategy followed to eliminate the items with correlated residuals that, as a result, defined a second factor when exploring the one-dimensionality of the factor in which they were loaded, together with an improvement in convergent validity (certainty that the items measure the same construct), ensures eliminating redundant items and poor indicators [47,51]. The factors with the largest item reduction were family support and friend support (from 15–6), which went from excellent reliability (λ_2_ > 0.90) to good (λ_2_ > 0.85). Importantly, all reliability coefficients inflate as the number of indicators increases, even if they are poor [54]. This slight loss in reliability was precisely due to the loss in the number of items, not to the loss of quality in the items because their measurement weights, the AVE, and even some of the correlations of concurrent validity increased. A detailed examination of the content of the remaining items showed they were reduced to their most essential or core part, yielding a shorter and more efficient scale. In the lack of support factor, which was reduced from seven to four items, the eliminated items corresponded to nonspecific content (for example, item 29 “In my family, we don’t talk about our shortcomings and mistakes”), and in the religious support factor, no items were eliminated. The four items of the neighbor support factor (which were not in the original version reported by Nava-Quiroz [31]), of which only three made explicit reference to neighbors, were all eliminated because they were absorbed in the religious support factor.

A strong finding supporting a four-factor reproducible structure with the 20 items retained from the SSNS is the convergence of the most reliable empirical criteria to determine the number of factors in both samples A (exploration) and B (of validation) as in the total sample. These criteria include Horn’s [49] parallel analysis, Velicer’s [62] minimum average squared partial correlation test, Ruscio and Roche’s [63] comparison data approach, the optimal coordinates method of Raiche et al. [64], and the new empirical Kaiser criterion from Braeken and van Assen [48].

Another concern that might arise regards the repeatability of the results in a random sample drawn from the same population. Although the sampling procedure was not probabilistic, when probabilistic sampling would be the most suitable [65], the sample collection setting (parent who regularly cares for the child in the hospital) and the large sample size guarantee that the population of the parent who is the main caregiver of a child with cancer admitted for treatment in the hospital is well represented. Consequently, the inferences derived from the present study should be limited to this population, and they would only be hypotheses in related populations. It should be noted that a probability sampling would also have this restriction [66].

The second objective of the study was to verify the reliability of the internal consistency of the scale. The expectation was excellent reliability for the 45 items of the scale, 12 items for friend support, and 12 items for family support [32,33]. This expectation was met. However, reliability downgraded to good after items were removed when redefining the socio-family support scale with 16 items and both factors (friend and family) with six items. The expectation of acceptable reliability was also met with the four items of religious support. The expectation of acceptable reliability for the seven items pertaining to lack of support was met, and the scale was kept for further reduction. The expectation of acceptable reliability for neighbor support was not met. Reliability for this factor was questionable, especially because the Cronbach’s alpha coefficient (Guttman’s lambda-3) was less than 0.60. It should be noted that in this study, Guttman lambda-2 and McDonald’s ω coefficients were used when the assumption of tau-equivalence was not met, that is, items having homogeneous true covariances [54].

The third objective was to describe the distribution of the scale and its factors. Consistent with expectations [32,34], no distribution followed a normal probability model; all presented negative asymmetry. This is because the majority of caregivers reported receiving a lot of or enough support (short interval from 5–3.4 in which many scores were concentrated), and very few indicated a lack of support (long interval from 3.99–1 in which few scores were dispersed). The strongest support came from the family (“a lot” as the central value) and religion (“quite a bit” as the central value). The central value of the friend support factor was the ambiguous response (neither agree nor disagree), and its distribution was the least negatively skewed; however, 40.1% of the participants said they received a lot of or quite a bit of support from friends, compared to 21.4% with very little or little support. Among these family caregivers, the importance of support can be ordered according to frequency as family, religious, and friend support, as there is a significant difference in the central tendency between these factors. This order of importance reflects cultural traits. In Mexico, the family occupies a central role; in turn, religion, with a clear predominance of Catholicism, is also very important in the socioemotional and spiritual lives of Mexicans. In contrast, friends occupy third place, especially in situations of crisis or need [67].

The fourth objective was to provide evidence of concurrent validity in relation to family functioning [17,33], resilience [35], and quality of life [36]. Significant correlations were expected with these three variables. These expectations were met. The SFS-16 and its factors of family support and lack of support correlated strongly with the total score for family functioning and moderately with the total scores for resilience and quality of life. The correlation of the friend support factor was medium with family functioning and low with the other two scales. Religious support was independent of these three total scores. The religious support scale only had positive correlations with support factors and social competence factors of the resilience scale and negative correlations with the environment and physical health factors of the quality of life scale. The worse the quality of life in environmental and physical aspects and the greater the social support and social competence, the more religious support is sought. Importantly, the correlations of RSS-4 with quality of life are opposite in sign of SFS-16 and its three factors (inverted scores in lack of support). As Bustamante et al. [68] point out, socio-family support seems to improve quality of life; that is, a better quality of life is a consequence of greater socio-family support. In the case of religious support, poor quality of life behaves as a cause or antecedent, as revealed by studies with people with chronic diseases [36,69] and older adults [70].

As previously noted, a limitation of the study is the use of nonprobabilistic sampling; therefore, inferences should be made with due caution among the main family caregiver of children with cancer treated in a public children’s hospital in Mexico City. This population is mainly made up of women, young people, and married women, with secondary school education, housewives, who have no income of their own, and individuals who identify as Catholics. Another limitation is that the study design was ex post facto; consequently, no causal inferences can be made. Finally, the associations and structure of social support found are limited to the measurement instrument used, the SSNS by López-Peñaloza [32].

## 5. Conclusions

Among these family caregivers of children with cancer, the SSNS has four factors. Three factors are intercorrelated and define a scale of socio-family support. The fourth factor is independent and defines a scale of religious support. The neighbor support factor of the original scale is not defined, as it is absorbed within the religious support scale. The consistency of the socio-family support scale with 16 items and its family support and friend support factors with 6 items each is good, and the consistency of its lack of support factor and religious support scale with 4 items each is acceptable. The distributions are asymmetric, with most participants receiving support. The strongest support comes from the family, followed by religious support and then friend support. Greater socio-family support is strongly associated with better family functioning, and socio-family support is strongly associated with greater resilience and better quality of life; however, religious support is only weakly associated with worse quality of life in environmental and physical aspects, as well as resilience aspects of greater competence and social support.

This abbreviated scale, reduced to 20 items from the original 45 items, is recommended for family caregivers due to its reliability and validity properties, in addition to being more convenient when applying a wide battery of measuring instruments. Interpretative norms of the scale should be set through percentile scores, using a probabilistic sampling. For this purpose, it should be checked which sociodemographic variables have an effect size that is at least medium on the scores in the scale and its four factors to set different norms based on the categories of these variables (for example, civil status: “married” versus “separated/divorced/widowed”; gender: “woman” versus “man”). Values below the 20th percentile could be interpreted as a low level of support. The present data suggest that greater socio-family support results in a better quality of life but that religious support is sought during times of worse quality of life, which requires further research.

## Figures and Tables

**Table 1:** Summary statistics of sociodemographic and clinical variables.

Sociodemographic and Clinical Variables	Value Labels	Caregiver				Child			
		*n*	%	M	SD	*n*	%	M	SD
Sex	Woman	515	81			302	48		
	Man	118	19			331	52		
Age (years)	1–2			31.70	7.58	226	35.7	5.75	4.92
	3–10					279	44.1		
	11–17					128	20.2		
	18–29	266	42						
	30–39	260	41.1						
	40–52	107	16.9						
Civil status	Married	257	40.6						
	Cohabitant	244	38.5						
	Divorced/separated	71	11.2						
	Single	55	8.7						
	Widowed	6	1						
Number of children	1	172	27.2	2.32	1.17				
	2	202	31.9						
	3	183	28.9						
	4–10	76	12						
Scholarship	Unschooled	18	2.8						
	Primary	124	19.6						
	Lower secondary	282	44.5						
	Upper secondary	163	25.8						
	Higher education	46	7.3						
Occupation	Housewife	413	65.2						
	Office employee	87	13.7						
	Merchant	58	9.2						
	Unemployed	44	7						
	Manual worker	26	4.1						
	Student	5	0.8						
Income	From 0–1	390	61.6						
(minimum wages) *	From 1–2	140	22.1						
	From 2–3	85	13.4						
	From 3–4	9	1.4						
	From 4–6	6	0.9						
	From 6–8	2	0.3						
Hospitalization time	≤1 week					392	61.9		
	≤1 month					148	23.4		
	<6 months					50	7.9		
	<2 years					20	3.2		
	≥2 years					23	3.6		
Diagnosis	Leukemia					280	44.2		
	Lymphoma					157	24.8		
	CNS tumors					83	13.1		
	Rhabdomyosarcoma					44	6.9		
	Neuroblastoma					33	5.2		
	Wilms’ tumor					20	3.2		
	Retinoblastoma					13	2.1		
	Bone tumors					3	0.5		

Note. *n* = absolute frequency, % = percentage, M = arithmetic mean, and SD = standard deviation. * Minimum wage = MXN 185.56 per day ≈ USD 7.72.

**Table 2:** Fit indices for 4 models for the Social Support Networks Scale (SSNS).

χ^2^	χ^2^	df	*p*	χ^2^/df	RMSEA (90% CI)	TLI	CFI	GFI	AGFI	SRMR
1. Five factors [32]. Subsample A (*n* = 316)										
	2537.851	935	<0.001	2.714	0.074 (0.070, 0.077)	0.742	0.756	0.693	0.652	0.073
2. Four factors with 33 items (EFA). Subsample A (*n* = 316)										
	1441.219	489	<0.001	2.947	0.079 (0.074, 0.083)	0.806	0.820	0.773	0.739	0.063
3a. Three factors for socio-family support with 16 items (CFA). Subsample A (*n* = 316)										
	213.231	101	<0.001	2.111	0.059 (0.048, 0.071)	0.931	0.942	0.926	0.901	0.055
3b. Single-factor model with 4 indicators for religious support. Subsample A (*n* = 316)										
	4.792	2	0.091	2.396	0.067 (0, 0.146)	0.975	0.992	0.993	0.964	0.022
4a. Three factors for socio-family support with 16 items. Subsample B (*n* = 317)										
	275.856	101	<0.001	2.731	0.074 (0.064, 0.085)	0.909	0.923	0.903	0.870	0.056
4b. Single-factor model with 4 indicators for religious support. Subsample B (*n* = 317)										
	0.378	2	0.828	0.189	0 (0, 0.066)	1	1	0.999	0.997	0.006

Note. Indices: χ2 = likelihood ratio chi-square statistic; df = degrees of freedom; *p* = probability value under the null hypothesis (H0): χ2 = 0; χ2/df = relative chi-square; RMSEA = point estimation for root mean square error of approximation and 90% confidence interval (CI) estimation, NFI = normed fit index, CFI = comparative fit index; GFI = goodness-of-fit index, AGFI = adjusted goodness-of-fit index; and SRMR = standardized root mean square residual.

**Table 3:** Reliability, convergent validity, and discriminant validity.

Models	NI	Sample						
		Total (*n* = 633)	B (*n* = 317)					
		λ_2_	ω	AVE	r^2^_Fi,F2_	r^2^_Fi,F3_	r^2^_Fi,F4_	r^2^_Fi,F5_
López-Peñaloza [32]	45	0.926						
F1: Friend support	15	0.913	0.912	0.414	0.236	0.099	0.017	0.908
F2: Family support	15	0.921	0.924	0.456		0.346	0.008	0.238
F3: Lack of support	7	0.703	0.698	0.272			<0.001	0.079
F4: Religious support	4	0.775	0.779	0.474				0.027
F5: Neighbor support	4	0.606	0.576	0.269				
EFA—4 factors	33	0.908						
Adjusted model: 4 factors	20	0.846						
SFS-16: 3 factors	16	0.875						
F1: Family support	6	0.881	0.888	0.571	0.130	0.293		
F2: Friend support	6	0.865	0.881	0.554		0.053		
F3: Lack of support	4	0.717	0.714	0.387				
Single-factor model for religious support	4	0.775	0.781	0.476				

Note. Statistics: NI = number of items, λ2 = Guttman’s lambda-2 coefficient, ω = McDonald’s omega coefficient, AVE = average variance extracted, and r^2^ = shared variance between the factors Fi (per row) and F2, F3, F4, or F5. Models: López-Peñaloza [32]: friend support (items 2, 5, 6, 10, 13, 17, 18, 27, 30, 31, 34, 38, 39, 42, and 45), family support (items 1, 3, 7, 8, 9, 12, 14, 20, 21, 33, 37, 40, 41, 43, and 44), lack of support (items 15, 22, 23, 25, 26, 29, and 35), religious support (items 4, 16, 28, and 36), and neighbor support (items 11, 19, 24, and 32). EFA: 4 factors (items 1, 3, 4, 7, 8, 9, 12, 14, 15, 16, 18, 21, 22, 23, 24, 25, 26, 27, 28, 30, 31, 33, 34, 35, 36, 37, 38, 39, 41, 42, 43, 44, and 45). Adjusted model—4 factors (items 1, 4, 8, 9, 14, 15, 16, 18, 21, 23, 25, 26, 28, 30, 31, 34, 36, 38, 39, and 43). Model 3 factors: family support (items 1, 8, 9, 14, 21, and 43), friend support (items 18, 30, 31, 34, 38, and 39), and lack of support (items 15, 23, 25, and 26). Single-factor model for religious support (items 4, 16, 28, and 36).

**Table 4:** Descriptive statistics and structure loadings of items.

Items	Statistics		Structure Loadings			
	M	SD	FAS	FRS	RS	LS
1. My family cares for me as I care for them.	4.263	0.975	0.712			
3. In my family, we are all equally important.	4.525	0.806	0.695			
4. My faith in God helps me overcome any difficulty.	4.351	0.855			0.630	
7. My family trusts me, and I trust them.	4.411	0.802	0.671			
8. I feel supported by my parents.	4.218	1.095	0.668			
9. Our family is very close.	4.174	1.020	0.788			
12. I have my partner’s support.	4.095	1.261	0.470			
14. We talk about problems in our family.	4.196	0.985	0.727			
15. When a problem comes up in my family, we solve it violently.	1.595	0.926				0.466
16. When I have problems, I go to church.	3.266	1.112			0.510	
18. My friends and I get together to talk, go to parties, go to the movies, etc.	2.987	1.171		0.629		
21. In my family, we encourage each other to improve ourselves.	4.222	0.916	0.752			
22. My friends and I know very little about each other.	2.722	1.095				0.523
23. My family and I cannot spend much time together because we fight.	2.101	1.161				0.632
24. My friends support me when I need them.	3.592	1.036		0.555		
25. There is not enough trust in my family.	2.196	1.232				0.455
26. It is difficult to work with friends because we end up fighting.	2.203	1.091				0.596
27. At work, most of my colleagues and I form a team to stay ahead.	3.623	1.005		0.500		
28. Prayer groups help me overcome things.	3.649	1.177			0.715	
30. My friends and I know each other very well.	3.294	0.972		0.680		
31. Outside of work, my colleagues and I get together to go out.	3.098	1.051		0.683		
33. When someone gets sick in my family, everyone worries.	4.373	0.963	0.708			
34. My friends and I visit each other’s houses.	3.307	1.091		0.694		
35. At work, we hide information that is useful for everyone.	2.373	1.017				0.457
36. My religious beliefs help me overcome any problem.	3.899	1.079			0.850	
37. Living within my family is excellent.	3.949	0.914	0.742			
38. My friends seek each other to discuss our problems.	3.446	1.011		0.740		
39. At work, we lend each other books, articles, etc.	3.089	1.041		0.651		
41. In my family, we are very communicative with each other.	3.953	1.027	0.727			
42. My friends and I think friendship comes first.	3.589	0.944		0.689		
43. It is normal to speak openly in our family.	4.028	0.934	0.756			
44. Despite our activities, we make time to spend as a family.	4.142	0.927	0.740			
45. My friends and I think that our friendship is valuable.	3.788	0.954		0.813		
Number of items			13	10	4	6
Guttman’s λ_2_			0.924	0.887	0.774	0.721

Note. Extraction method: maximum likelihood. Rotation method: Promax. Subsample A (*n* = 316). FAS = family support, FRS = friend support, RS = religious support, and LA = lack of support. Statistics: M = arithmetic mean, and SD = standard deviation.

**Table 5:** Descriptive statistics and normality tests.

Statistics	SFS-16	FAS-6	FRS-6	LS-4	RSS-4
[1, 1.8)	0.5%	3%	6.6%	47.4%	2.7%
[1.8, 2.6)	4%	1.4%	14.8%	31.3%	6.3%
[2.6, 3.4)	21.8%	7%	38.4%	14.7%	19.1%
[3.4, 4.2)	51%	34.4%	32.4%	5.7%	36.3%
[4.2, 5]	22.7%	54.2%	7.7%	0.9	35.6%
M	3.756	4.176	3.166	1.989	3.773
SD	0.615	0.790	0.844	0.810	0.822
Mdn	3.813	4.333	3.167	2	4
SIQR	0.406	0.500	0.500	0.625	0.500
Z_Sk_	−6.938	−17.711	−3.557	7.361	−7.907
Z_K3_	4.376	19.644	0.546	1.165	2.582
K_2_	67.290 ***	699.591 ***	12.949 ***	55.539 ***	69.193 ***
Max(│D│)	0.060 ***	0.148 ***	0.098 ***	0.123 ***	0.132 ***
P10	3.000	3.333	2	1	2.750
P20	3.250	3.667	2.5	1	3
P25	3.375	3.833	2.667	1.250	3.250
P30	3.500	4	2.833	1.500	3.500
P40	3.625	4.167	3	1.750	3.750
P50	3.813	4.333	3.167	2	4
P60	4.000	4.500	3.500	2	4
P70	4.125	4.667	3.667	2.250	4.250
P75	4.188	4.833	3.667	2.500	4.250
P80	4.250	4.833	3.833	2.750	4.500
P90	4.438	5	4.167	3	4.750

Note. Total sample (*n* = 633); M = arithmetic mean; SD = sample standard deviation; Mdn = median; SIQR = semi-interquartile range; ZSk = standardized value of sample skewness based on the third central moment; ZK3 = standardized value of sample kurtosis excess based on the fourth central moment; K2 = D’Agostino–Pearson normality test statistic; Max(∣D∣) = Kolmogorov–Smirnov normality test statistic with Lilliefors correction for probability value. *** = probability value under null hypothesis of normal distribution < 0.001. P10 to P90 = percentile scores (deciles and quartiles). SFS-16 = socio-family support scale = (I1 + I8 + I9 + I14 + (6 − I15) + I18 + I21 + (6 − I23) + (6 − I25) + (6 − I26) + I30 + I31 + I34 + I38 + I39 + I43)/16. FAS-6 = family support factor = (I1 + I8 + I9 + I14 + I21 + I43)/6. FRS-6 = friend support factor = (I18 + I30 + I31 + I34 + I38 + I39)/6. LS-4 = lack of support factor = (I15 + I23 + I25 + I26)/4. RSS-4 = religious support scale = (I4 + I16 + I28 + I36)/4.

**Table 6:** Convergent validity.

Scale	Factor	λ_2_	SFS-16	FAS-6	FRS-6	LS-4	RSS-4
Family functioning scale		0.888	0.642 **	0.616 **	0.309 **	−0.600 **	0.020 ^ns^
	Positive family environment	0.789	0.584 **	0.579 **	0.304 **	−0.467 **	0.037 ^ns^
	Cohesion	0.804	0.536 **	0.597 **	0.251 **	−0.439 **	0.073 ^ns^
	Hostility	0.679	−0.394 **	−0.422 **	−0.156 **	0.409 **	−0.037 ^ns^
	Troubles with rules/feelings	0.695	−0.481 **	−0.368 **	−0.243 **	0.533 **	0.043 ^ns^
Quality of life scale	Quality of life scale	0.866	0.411 **	0.385 **	0.233 **	−0.316 **	−0.078 ^ns^
	Physical health	0.529	0.182 **	0.159 **	0.102 *	−0.123 **	−0.086 *
	Psychological health	0.568	0.330 **	0.341 **	0.174 **	−0.263 **	0.020 ^ns^
	Social relationships	0.680	0.418 **	0.357 **	0.258 **	−0.344 **	−0.039 ^ns^
	Environment	0.763	0.397 **	0.354 **	0.245 **	−0.310 **	−0.102 **
	General quality of life	-	0.385 **	0.407 **	0.174 **	−0.267 **	−0.006 ^ns^
	Health condition	-	0.346 **	0.330 **	0.182 **	−0.271 **	−0.014 ^ns^
Resilience scale		0.952	0.438 **	0.493 **	0.234 **	−0.329 **	0.070 ^ns^
	Strength and confidence	0.938	0.263 **	0.329 **	0.100 *	−0.231 **	−0.024 ^ns^
	Social competence	0.863	0.343 **	0.288 **	0.291 **	−0.226 **	0.103 **
	Family support	0.893	0.493 **	0.590 **	0.181 **	−0.391 **	0.061 ^ns^
	Social support	0.917	0.526 **	0.546 **	0.297 **	−0.377 **	0.125 **
	Structure	0.760	0.172 **	0.216 **	0.152 **	−0.033^ns^	0.070 ^ns^

Note. Total sample (*n* = 633). λ2 = Guttman’s lambda-2 coefficient. Probability value under null hypothesis of rS = 0 in a 2-tailed test: ns *p* > 0.05, * *p* < 0.05, ** *p* < 0.001. SFS-16 = 16-item socio-family support scale; FAS-6 = family support factor; FRS-6 = friend support factor; LA-4 = lack of support factor; RSS-4 = religious support scale.
